# Probiotic Functions in Fermented Foods: Anti-Viral, Immunomodulatory, and Anti-Cancer Benefits

**DOI:** 10.3390/foods13152386

**Published:** 2024-07-28

**Authors:** Yeonhee Pyo, Ki Han Kwon, Yeon Ja Jung

**Affiliations:** 1 Department of Beauty Cosmetics, College of Biomedical and Health Science, Konkuk University, Chungju 27478, Republic of Korea; 2 College of General Education, Kookmin University, Seoul 02707, Republic of Korea; kihan.kwon@kookmin.ac.kr

**Keywords:** fermentation, probiotics, nutritional improvement, human health

## Abstract

Fermented foods can provide many benefits to our health. These foods are created by the action of microorganisms and help support our digestive health and immune system. Fermented foods include yogurt, *kimchi*, pickles, kefir, beer, wine, and more. Fermented foods contain probiotics, lactic acid bacteria (LAB), yeast, organic acids, ethanol, or antimicrobial compounds, which help balance the gut microbiome and improve digestive health. Fermented foods can also benefit your overall health by increasing the diversity of your gut microbiome and reducing inflammation. By routinely consuming fermented foods with these benefits, we can continue to improve our health. Probiotics from fermented foods are beneficial strains of bacteria that are safe for human health and constitute an important component of human health, even for children and the elderly. Probiotics can have a positive impact on your health, especially by helping to balance your gut microbiome and improve digestive health. Probiotics can also boost your immune system and reduce inflammation, which can benefit your overall health. Probiotics, which can be consumed in the diet or in supplement form, are found in many different types of foods and beverages. Research is continuing to investigate the health effects of probiotics and how they can be utilized. The potential mechanisms of probiotics include anti-cancer activity, preventing and treating immune system-related diseases, and slowing the development of Alzheimer’s disease and Huntington’s disease. This is due to the gut–brain axis of probiotics, which provides a range of health benefits beyond the digestive and gastrointestinal systems. Probiotics reduce tumor necrosis factor-α and interleukins through the nuclear factor-kappa B and mitogen-activated protein kinase pathways. They have been shown to protect against colon cancer and colitis by interfering with the adhesion of harmful bacteria in the gut. This article is based on clinical and review studies identified in the electronic databases PubMed, Web of Science, Embase, and Google Scholar, and a systematic review of clinical studies was performed.

## 1. Introduction

Probiotics are live microorganisms that provide health benefits to their hosts when administered in appropriate amounts [1]. Probiotics have direct and indirect effects on health by altering the composition of the gut microbiome to produce bioactive metabolites [2]. The use of probiotics has been widely covered in the medical, pharmaceutical, and food sectors for years, and the consumption of probiotic products continues to grow due to the health benefits provided by microorganisms [3]. Probiotics are non-pathogenic microorganisms that, when administered in the right amounts, exert health benefits on their hosts and are finding new applications, especially in the food industry. They regulate the gut microbiome through dietary intake and is used as an adjunct for immune system enhancement, prevention of hypertension and hypercholesterolemia, cancer prevention, and gastrointestinal diseases [4]. And as functional foods, probiotic ingredients have become very important and are considered as potential microorganisms with health-promoting functions [5]. Dietary intake of probiotics is largely in the form of dairy or non-dairy products such as ice cream and yogurt, as food, beverages, and supplements [6]. Alternatively, the active microorganisms in fermented foods are genetically similar to the probiotic strains to promote bioactivity [7]. The species of safe probiotics are determining a safe probiotic species includes evaluating its safety and effectiveness, whether it has been used for a long period of time with a historical safety record, whether it has been proven safe by clinical trials, whether it has the potential to cause toxicity and side effects, whether it is genetically stable, and whether its safety is ensured by a defined production process [8,9]. For instance, *Lactobacillus acidophilus*, *L. Casei*, *L. Plantarum*, *L. Paracasei*, *L. Johnsonii*, *L. Rhamnosus*, *L. Reuteri*, *Bifidobacterium bifidum*, *B. Infantis*, *B. Lactis*, *Saccharomyces bourlardii*, and *Propionibacterium freudenreichii*, and in general, the probiotic species used by humans, belong to the *genera Bifidobacterium*, *Lactobacillus*, *Propionibacterium* and *Saccharomyces* [10].

These probiotics have an excellent masking effect and strengthen immunity from irritants, treat infections, improve symptoms of irritable bowel syndrome and colitis, prevent colon cancer and cancer, and help prevent obesity [11].

Several neo-biotics supplements or nutraceuticals are now a mainstay in the nutrition industry, but they differ from the probiotics that are traditionally obtained from fermented foods. Fermented foods are part of the staple diet in many countries and are characterized by their efficient preparation. The probiotics ingested through them have been shown to influence vagal nerve activity in the microbiome–gut–brain axis [12]. Fermentation works by breaking down polypeptides into amino acids, which can improve the digestibility of proteins and carbohydrates in the body [13,14]. And it can improve calcium bioavailability for skeletal health by promoting the enrichment of key nutrients [15]. In these fermentation matrices, lactase enzymes produced by bacteria are present [16]. The *lactic acid bacteria* (LAB) species found in fermented foods are high producers of vitamins and antioxidants, and in the case of the *lactobacillus* family, have been implicated in the improvement of metabolic syndrome and anti-cancer and immune function, and the lowering of blood pressure and cholesterol [17,18,19,20,21]. Other bacterial probiotics, such as Propionibacterium and Bifidobacterium, are also produced in fermented foods [22]. These effects symbolize the compounds and bioactive functions produced during the fermentation process and suggest that fermented foods can have a positive impact on the gut microbiota and health in both the short and long term and can be considered an important component of the human diet [23]. The beneficial bacteria known as probiotics, derived from fermented foods, are classified into categories such as prebiotics, postbiotics, bacteriobiotics, and psychobiotics. These categories share similarities and differences among them, and are the focus of study in neobiotics [24].

Prebiotic substances from fermented foods are carbohydrate-based, with prebiotic effects exerted by polyphenols and polyunsaturated fatty acid substances, prebiotic substances that come from fermented foods. Fermented foods can contain carbohydrate-based substances that have prebiotic effects [25]. Additionally, postbiotics in fermented foods are less well known, but they can have a positive impact on the microbiome, inflammation, and gut physiology and are recognized by the international society for the science of probiotics and prebiotics as microorganisms or components that confer health benefits to the host [26]. Bacterial biotics from fermented foods also have the potential to be used for disease prevention purposes to move from traditional foods to functional foods [27]. In recent years, the use of psychobiotics has emerged as a way to treat chronic problems such as anxiety and depression, not only by improving intestinal function, but also by regulating immune, humoral, and neuronal metabolic pathways to exert anxiolytic and antidepressant effects. This has been discussed as a possible aid in maintaining and restoring population mental health, requiring an understanding of the sophisticated protocols that can be derived from food [28].

In this context, the bioactive food mechanisms associated with fermented foods recognize the benefits of fermentation as a health-promoting ingredient, with increased vitamin content, antioxidant, antidiabetic, and antihyperlipidemic activities [29]. Therefore, this review will analyze the fermentation pathways in food and review the health benefits of probiotics in terms of their physiological properties. This study aims to propose a safe and effective way to utilize probiotics in future fermented foods based on deep insights. In addition, we aim to observe the future trends in probiotic foods through a literature review and bibliometric analysis.

## 2. Fermentation Pathways in Foods

Representative fermented food and beverage products such as yogurt, *kimchi*, cheese, wine, beer, natto, etc., achieve the conversion of ingredients in food through the controlled growth of microorganisms and the action of microbial enzymes [30]. *Lactobacilli*, beneficial bacteria, are found in fermented foods. During this process, lactose is fermented to produce oxygen and lower the pH, making the food sour [31]. Lactic acid is the main organic acid in fermented milk and is degraded by LAB. These include formic and acetic acids and have the metabolic pathway of Embden–Meyerhof–Parnas (EMP) [32]. The EMP pathway is a linear process that converts glucose to lactate by breaking down fibrulose into lactic acid. It is responsible for producing, preserving, and creating an acidic environment for food, which gives it its characteristic tangy flavor [33]. Bacteria metabolize sugars by the phosphoketolase pathway. When degraded, pentose sugars typically produce small amounts of lactic acid, acetic acid, alcohol, and carbon dioxide. This can be seen in the fermentation of sourdough bread or in the fermentation of other foods [34,35].

The organic acids, ethanol, and antimicrobial compounds naturally found in foods have the ability to inhibit all *Pathogenic bacteria* and *Spoilage organisms* [36], and the consumption of probiotics is useful for maintaining a healthy gut microbiome against *Pathogenic bacteria*, which can help improve digestion and the immune system [37]. The fermentation pathway of food products results in changes in the nutritional and biochemical quality of the starting ingredients. It is a highly complex ecosystem of raw material enzymes interacting with the metabolic activity of fermenting microorganisms, resulting in benefits in terms of antioxidants, peptide production, organoleptic and probiotic properties, and antimicrobial activity compared to simple foods. The fermentation pathway is the change in biochemical compounds that determine the properties of the fermented food [38]. The fermentation pathway in food refers to the biochemical process in which *Microorganisms* metabolize nutrients in a food substrate, resulting in various changes such as the production of acids, alcohols, gases, and other metabolites [39]. Common fermentation pathways for food include the following:

First, lactic acid fermentation: This pathway is prevalent in dairy products such as yogurt, cheese, and kefir, and fermented vegetables such as *Kimchi*. LAB such as *Lactobacillus* and *Streptococcus* convert sugars into lactic acid, which produces the tangy flavor and acidic pH [40,41,42,43]. This pathway, used in the manufacture of alcoholic beverages such as ethanol-fermented beer, wine, and spirits, is determined by yeasts such as *Saccharomyces cerevisiae*. Yeast converts sugars into ethanol and carbon dioxide through anaerobic metabolism [44]. However, in lactic acid fermentation by bacteria such as LAB, using wine as an example, a variety of yeasts adhere to damaged grapes and convert p-coumaric acid to 4-ethylphenol [45,46]. This leads to the presence of phenolic off-odors, and as the concentration of 4-ethylphenol increases, unpleasant odors are produced by fermentation [47,48]. Another route is acetic acid-fermented vinegar. This can be found in fermented foods that are made through acetic acid fermentation. Acetic acid bacteria, *Acetobacter* and *Gluconacetobacter*, convert ethanol into acetic acid in the presence of oxygen, which gives vinegar its sour flavor [49]. Second, propionic acid fermentation: This pathway is mainly associated with the production of Swiss cheese. Propionic acid bacteria contribute to the flavor and texture of cheese by converting lactic acid and acetate into propionate, carbon dioxide, and acetate [50]. Propionic acid can improve food quality by regulating pH during fermentation [51,52], and its higher pH than *Clostridium* reduces lactose and accelerates popionic acid fermentation in Swiss-type cheese [53]. This is attributed to genomic shuffling and molecular mechanisms of acid-resistant mutant strains of Propionibacterium acidipropioni and changes in acid accumulation, resulting in increased propionic acid production through fermentation [54]. And acid tolerance mutations affect membrane proton pumps, glutamate decarbonylase, and arginine deaminase, which in turn affect cell wall structure [55,56]. And certain bacteria, such as butyric acid-fermenting *Clostridium* species, can perform butyric acid fermentation, which produces butyric acid and other volatile fatty acids. This pathway is involved in the fermentation of some types of cheese and pickles. Butyric and caproic acids have value as bioresources and hold promise for bioaugmentation strategies that can be used as products [57]. Finally, there is mixed acid fermentation. Some bacteria, such as certain strains of E. coli, use mixed-acid fermentation to produce a mixture of organic acids (e.g., lactic, acetic, and formic) as well as ethanol and gases (e.g., carbon dioxide and hydrogen) [58]. For the production of short-chain fatty acids by gut microbes, probiotics have metabolite formation mechanisms that regulate the production of volatile fatty acids (SCFAs) [59]. SCFAs are the carbon flux from the diet to the host microbiome [60], with acetic, propionic, and butyric acids being the most common. The fermentation of rapidly fermentable non-digestible carbohydrates produces another organic acid—lactic acid [61]. Although this acid does not belong to the SCFA group, it can be produced by LAB, e.g., the genera *Lactobacillus* and *Bifidobacterium* [62].

These fermentation pathways play an important role in food preservation, flavor development, and texture enhancement. In particular, the specific pathway used depends on factors such as the microorganisms involved, the composition of the food substrate, environmental conditions (pH, temperature, and oxygen availability), and the desired end product [63,64].

## 3. Mechanisms of Health Benefits of Fermented Foods

Fermented foods can also help with the synthesis of certain vitamins and the regulation of the immune system, which can reduce the risk of gastrointestinal disorders, allergies, and inflammatory conditions [65,66]. Probiotics downregulate the production of pro-inflammatory cytokines and prevent apoptosis. They inhibit T cell proliferation and prevent various inflammatory events from occurring [67]. They also produce hydrogen peroxide for inhibition of bacterial vaginosis or related pathogens [68] and have the ability to survive in both acidic and alkaline environments in the gastrointestinal tract [69]. Along with probiotics, postbiotics are also known as useful bioactive substances. Postbiotics, produced by live LAB during the fermentation process, enhance the health benefits of food products by positively modifying their pro-health value. This includes components such as short-chain fatty acids, enzymes, peptides, and other bioactive compounds that contribute to improved gut health and immune function [70]. They are produced during fermentation in a matrix and are useful for improving immunity and treating food allergies in infants and children.

**Figure 1:**
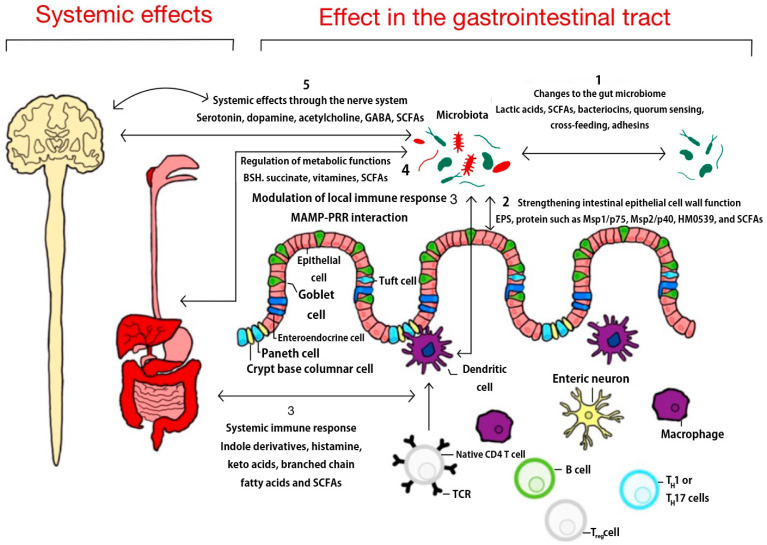
Gut biology of probiotics. SCFAs, short-chain fatty acids; GABA, γ-aminobutyric acid; Treg, regulatory T lymphocyte; MAMP-PRR, microbe-associated molecular patterns pattern recognition receptors; EPS, exopolysaccharide.

They are composed of carbohydrate elements with peptide, teichoic acid, and plasmalogen structures and are classified on the basis of various complex molecules such as p40 and p75 molecules, lactose, B vitamins, proteins of butyrate, acetate, lactate, and propionate, and organic acids 3-phenylactic acid and propionate. They are promising therapeutic agents for the prevention of gastrointestinal diseases and are considered as therapeutic technologies due to their mild side effects [71]. These mechanisms are illustrated in Figure 1.

Probiotics strengthen the function of the intestinal cell walls by colonizing the gut with more beneficial bacteria than harmful bacteria. They regulate the immune response and have the effect of treating systemic diseases, including metabolic and nervous systems. And because they are not killed by stomach and bile acids, they can be transported to the heart, cells, and more [72,73]. These probiotics and postbiotics have disease-modifying properties. The process of modulating the gut microbiota and metabolic pathways in the context of ulcerative colitis, especially postbiotics, is free from safety concerns and modulatory effects. They have the potential to be the next generation of biotherapeutics and are emerging as a treatment for colitis and other diseases [74]. As such, biotics are a therapeutic pathway for disease, and interactions between the gut microbiome and the central nervous system (CNS) have been implicated in a variety of neurodegenerative diseases, developmental disorders, and mood regulation [75,76]. Bacteria in the gut produce acetate, propionate, and SCFA to facilitate communication between the brain and gut via immune cells and cytokines [77]. They also produce indole and its derivative tryptophan metabolites and microbial neurotransmitters γ-aminobutyric acid (GABA), serotonin, and catecholamines. Depending on the composition of the gut microbiome, this will either balance dysbiosis or achieve a protective function against disease [78,79,80].

## 4. Health Benefits of Fermented Probiotics

Probiotics are living organisms that are believed to cause health benefits to their hosts without causing harm. The clinical efficacy of probiotics has been shown in the treatment or prevention of some gastrointestinal inflammation-related conditions, including traveler’s diarrhea, antibiotic-associated diarrhea, pouchitis of the restorative ileal pouch, and necrotizing enterocolitis [81]. Probiotics have been shown to improve immune function by promoting the growth of beneficial bacteria in the body and encouraging the fight against harmful pathogens. In particular, the gut microbiome has been shown to play an important role in the immune system [82,83,84]. Anti-inflammatory properties have also been found to reduce inflammation by some strains of biotics. They may be a beneficial preventive and therapeutic tool for patients with inflammatory diseases such as Crohn’s disease, ulcerative colitis, and arthritis [85,86,87]. And with a two-way communication system on the gut–brain axis, probiotic intake may improve mental health by reducing symptoms of depression, anxiety, and stress [88,89,90]. In terms of nutrition, probiotics can produce enzymes that aid in the digestion and absorption of nutrients such as vitamins and minerals, and have weight management benefits, reducing body fat and supporting weight management through appetite control [91]. They also prevent antibiotic-associated diarrhea, which can cause diarrhea and other gastrointestinal symptoms through the disruption of the balance of gut bacteria. This may reduce the risk of diarrhea as probiotic intake during or after antibiotic therapy restores the balance in the gut [92,93,94].

To map the current state of knowledge on the association of probiotics in fermented foods with various health benefit functions, key findings from each article were extracted and transcribed into a table (Table 1). The analysis included comparing and contrasting between papers, identifying and organizing categories of findings, and summarizing the categories overall.

The health benefits of probiotics from fermented foods for digestive health were shown in a clinical trial of 52 women aged 20–55 years old with irritable bowel syndrome (IBS). A probiotic diet of Fermentable oligosaccharides, Disaccharides, Monosaccharides, and Polyols has been proven to alleviate IBS symptoms and improve patients’ quality of life [95,96]. In addition, a clinical trial of 71 patients randomized to probiotics or probiotics and prebiotics in a blinded trial compared to placebo confirmed the effectiveness of probiotics and probiotics and prebiotics in improving microbial genes. After achieving human immunodeficiency virus (HIV)-1 suppression with combination antiretroviral therapy, HIV-1-infected patients have difficulty fully restoring normal CD4+ T cell counts, resulting in immune disharmony. The positive effects of probiotics have been demonstrated [97]. In addition, randomized clinical trials have demonstrated the impact of probiotics on the gastrointestinal tract for *H. pylori* control and adverse events. A randomized controlled trial of a probiotic (*Bifidobacterium* tetragenous viable bacterium; tetragenous bacterium) or placebo supplementation showed the depletion of *H. pyloriand* neutralization of *Bacteroidetes*, and the effect of probiotics on the eradication of *H. pyloriand* alleviation of adverse effects in the trial suggested that changes in the GI microbiome may be modulated by probiotics [98].

When it comes to the immune system, probiotics have been shown to improve immunity. This was found in a probiotic intervention trial involving 187 pregnant women. Clinical trials using probiotic-enriched colostrum have shown that probiotics may improve maternal immune system and infant immune function [99]. It has also been shown to have a positive effect on preventing pregnancy complications in pregnant women [100]. In children, it is also possible to induce beneficial changes in gut microbiome (GM) composition and gene function. This has been demonstrated in a randomized clinical trial in children [101] for the clinical effectiveness of probiotic supplementation for diarrhea prevention.

The effect on inflammation has been demonstrated by probiotics as an effective adjunctive treatment for Alzheimer’s disease (AD) due to their modulatory effect on the gut–brain axis. This was a trial that demonstrated the independent impact of probiotics, with the clinical results showing significant improvements in serum markers of inflammation and oxidative stress, which could have a beneficial impact on patients’ quality of life and physical activity [102]. Probiotics also hold promise as an oral treatment for patients with chronic kidney disease (CKD), especially in hemodialysis. This can increase the expression levels of miR-29a and miR-29b, which may improve malnutrition and reduce tumor necrosis factor-α (TNF-α) as an oral nutritional agent for chronic kidney disease [103]. The effect of probiotics in reducing natural killer T cells (NKT cells) and inflammatory factors in children with pneumonitis is accompanied by a protective effect on long-term lung function. It also significantly reduces inflammatory factors such as levels of interleukins (IL)-4 and IL-10, interferon-γ, and immunoglobulin E, and also increases the percentage of NKT cells in the blood, resulting in a more positive effect than placebo [104].

There are modulators of the microbiome–gut–brain axis (MGBA) called psychobiotics that have promising benefits for mental health. This has been demonstrated in trials of the effects of probiotic supplementation on well-being and quality of life, emotion regulation, mindfulness, and anxiety perception. This in turn suggests that the interaction of biotics intake with health behaviors may provide positive effects on anxiety, emotion regulation, and mindfulness [105]. The health properties of probiotics are also involved in bone metabolism. This was validated in a randomized, double-blind, placebo-controlled trial to evaluate the effects of multispecies probiotic supplementation. Experiments showed a significant decrease in the median serum bone resorption marker C-terminal telopeptide of type I collagen, suggesting that probiotics may improve bone health through anti-resorptive effects in postmenopausal women [106].

*Lactobacillus*, a type of probiotic, plays a role in the absorption of iron intake. Pregnant women, in particular, are at risk of iron deficiency despite iron intake recommendations, and *lactobacillus* may help to replenish deficiencies by facilitating iron absorption [107].

Probiotics, along with prebiotics, have also been shown in clinical trials to be effective in the management of obesity. They have shown positive results in weight loss and psychological impact in obese patients, with significant reductions in insulinemia and homeostatic model assessment for insulin resistance (HOMA-IR). Dietary probiotics have also been shown to reduce fasting blood sugar and improve depression, anxiety, and stress [108].

Probiotics also have the potential to reduce risk factors for cardiovascular disease (CVD), including variable cholesterol and body mass index (BMI). The detection of glycemic reduction and antioxidant effects was greater in overweight volunteers, suggesting a role for probiotics in obesity management in CVD patients. Also, the potential probiotic *Lactiplantibacillus plantarum inducia* strain has been shown to express antioxidant effects on blood lipids and implications for the application of dietary probiotic supplementation for CVD risk management [109].

**Table 1:** Clinical trials of probiotics for health benefits.

Subjects	Health Benefit	Setting	Findings	Reference
52 female patients with IBS	Digestive	Monitored individuals for 6 weeks Participants completing the Hospital Anxiety and Depression Scale, IBS-QOL, and IBS Symptom Severity Score (IBS-SSS) at the beginning and end of the study	FODMAP diet benefits people by reducing the severity of IBS symptoms and improving the quality of life.	[96]
71 patients with HIV-1 infection	Digestive Immunities	Randomized controlled trial Randomly assigned to an active dietary intervention	6 + 3 month bioassessment with 1:2:2 randomization of placebo, probiotic, and probiotic plus prebiotic.	[97]
276 untreated *H. pylori* positive patients	Gastrointestinal	Double-blinded Randomized trial Two groups receiving probiotics and placebo; 14 days of quadruple bismuth therapy for 28 days; saliva, gastric mucosa, and feces samples collected before and after treatment for 16S rRNA gene sequencing	Treatment with probiotics resulted in smaller changes in microbiome compared to placebo. Oral expansion of some pathogenic genera, including *Porphyromonas* and *Leptotrichia*, was inhibited by probiotics.	[98]
187 pregnant women	Colostrum immune system of infant	Randomized in a double-blind manner into four intervention groups	Probiotic intervention affects colostrum immune mediator concentrations. Probiotic intervention in colostrum contributes to infant immune development.	[99]
72 pregnant women	Immune system	Double-blinded randomized controlled trial Bifidobacterium longum subsp. *B. longum* 1714; daily intake of at least 1 × 109 colony-forming units or placebo in healthy pregnant women 16 to 20 weeks gestation until delivery	Manipulation of the maternal immune response by probiotics has the potential to reduce pregnancy complications.	[100]
70 children	Preventing diarrhea in children	Randomized-controlled trial Double-blinded, placebo-controlled study design Intervention group (IG, *n* = 35) which received conventional treatment plus the probiotic, and a control group (CG, *n* = 35)	Safe and effective as an adjunctive treatment for pediatric acute watery diarrhea. Effective in reducing the duration of diarrhea. Induces beneficial changes in GM composition and gene function.	[101]
90 patients with AD	Improved serum inflammation and oxidative stress	12-week placebo-controlled, double-blind, randomized clinical	Significant improvements in serum inflammation and oxidative stress markers were observed. Probiotic supplementation for 12 weeks had beneficial effects on oxidative stress, inflammation, quality of life, and physical activity in patients with mild to moderate AD.	[102]
31 patients with CDK	Anti-inflammatory and anti-fibrotic properties	Conducted a randomized, multicenter, parallel-group trial using three groups: 1: control (C) individualized diet (*n* = 11); 2: ONS + ONS-PL (*n* = 10); 3: ONS-PR (*n* = 10)	Probiotics increase the expression levels of miR-29a and miR-29b, suggesting that they regulate and modify target gene expression with anti-inflammatory and anti-fibrotic actions. There are potential benefits of probiotics for malnourished patients with chronic kidney disease on hemodialysis.	[103]
100 children with sepsis	Improve long-term prognosis	Randomly divided into a placebo and probiotic group, with 50 people in each group	Proportion of NKT cells in blood was gradually reduced by probiotics. Percentage of peripheral blood NKT cells in the probiotic group was significantly lower than placebo. Probiotics reduce inflammatory factors.	[104]
135 adults	Mental health	Randomized placebo-controlled clinical trial	Probiotic intake has effects on well-being, quality of life, emotion regulation, anxiety, mindfulness, and interoceptive awareness. Lifestyle behaviors can be controlled for in psychobiotic and mental health studies.	[105]
40 women with postmenopausal osteopenia	Bone metabolism	Randomized, double-blind, placebo-controlled trial	Multispecies probiotics may have a preventive effect on bone through anti-resorptive effects in postmenopausal women.	[106]
20 pregnant women	Iron absorption	Double-blind, placebo-controlled, randomized supplementation feasibility study	Influence on maternal and neonatal hematologic and iron status. Probiotics supplement maternal hematologic and iron status throughout pregnancy compared to the placebo group.	[107]
45 obese patients	Obesity management	Clinical trials Observed 1 month after intervention Perform anthropometric measurements, biological parameters, dietary surveys, and psychological scores	Probiotics and prebiotics have been shown to prevent obesity, improve obesity-related mental illness, and manage sarcopenic obesity.	[108]
240 adults	Antiglycemic Antioxidant	Two parallel-armed double blind trials	Probiotics may reduce CVD risk, and indusial strains may benefit cardiovascular health by regulating blood lipids and blood sugar.	[109]

FODMAP, fermentable oligosaccharides, disaccharides, monosaccharides, and polyols; IBS, irritable bowel syndrome; IBS-QOL, IBS quality of life scale; IBS-SSS, IBS symptom severity score; GM, gut microbiome; AD, Alzheimer’s disease; ONS, oral nutritional supplement; ONS-PL, ONS placebo; ONS-PR, ONS + probiotics; NKT, natural killer T; CVD, cardiovascular disease.

## 5. Disease and Probiotics

Probiotics can improve digestive health and help prevent and treat digestive diseases. In particular, they can help alleviate symptoms of digestive conditions such as chronic inflammatory bowel disease and irritable bowel syndrome [110]. Probiotics also have anti-cancer effects; therefore, probiotic strains have potential mechanisms of anti-cancer activity, as they can be used as chemotherapeutic agents for preventive cancer treatment [111]. In addition, in people with oncologic syndromes, probiotics maintain gut microbial homeostasis and alter immunologic and cellular responses by increasing the epithelial barrier, which leads to anti-inflammatory, antioxidant, and anti-cancer effects that reduce cancer burden and growth. This may be a mechanism for therapeutic strategies based on the role of probiotics [112]. This suggests that probiotics may help prevent the development of cancer or inhibit the growth of tumors [113]. Various inhibitory effects of probiotics on colorectal cancer cells at different concentrations and times were identified, and drug combinations with probiotics showed therapeutic effects, with survival of HT-29 cells as low as 38% [114]. Probiotics also have a positive impact on type 2 diabetes (T2D). They can reduce chronic systemic inflammatory conditions, insulin resistance, waist circumference, and improve glycemic profiles. The addition of multi-strain probiotics improves β-cell function in T2D patients [115]. The role of probiotics in AD is also important. Probiotics have been shown to improve cognitive function due to their role in neurotrophic factors and oxidative stress in the pathogenesis of AD. A clinical trial in AD patients showed a 36% increase in brain-derived neurotrophic factor (BDNF), a decrease in IL-1β, and an increase in the antioxidant superoxide dismutase (SOD), which may improve cognitive function [116]. Probiotics also alleviate the symptoms of Huntington’s disease (HD), which is characterized by a decreased quality of life due to gastrointestinal symptoms. Probiotics have been shown to have positive outcomes for gut dysbiosis and neurodegenerative diseases. This implies that the potential utility of the gut as a therapeutic target in HD is closely linked to probiotics [117].

Probiotics have a wide range of health benefits, including the prevention and treatment of cancer, the prevention and management of diabetes, and a role in reducing the risk of Alzheimer’s disease and improving cognitive function, and the anti-inflammatory and antioxidant effects of gut microbes may slow the progression of HD and contribute to symptom relief.

**Figure 2:**
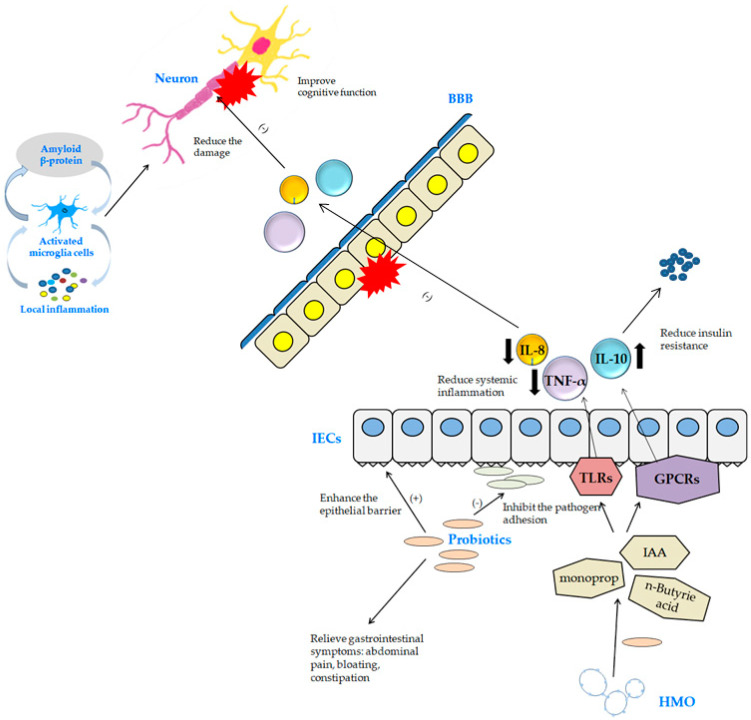
Potential mechanisms of probiotics. HMO, human milk oligosaccharides; SCFA, short-chain fatty acids; GPCRs, G protein-coupled receptors; APC, antigen-presenting cells; IECs, intestinal epithelial cells; NF-κB, nuclear factor kappa-light-chain-enhancer of activated B cells; MAPK, mitogen-activated protein kinase; IL-10, interleukin 10; IL-8, interleukin 8; TNF-α, tumor necrosis factor α; BBB, blood–brain barrier; IAA, auxin; TLRs, Toll-like receptors.

The role of probiotics in cancer prevention and treatment is particularly prominent in colorectal cancer. Probiotics have been shown to reduce the risk of colorectal cancer and inhibit tumor growth [118]. Most probiotics are part of the natural gut flora and include *Lactobacillus*, *Bifidobacteria*, *Lactococcus*, *Streptococcus*, *Enterococcus*, Gram-positive bacteria belonging to the genus Bacillus, and some strains of yeast belonging to the genus *Saccharomyces* [119]. These probiotics can directly modulate and improve the host’s gut microbiota as a novel colorectal cancer treatment modality [120]. And for the prevention and treatment of cancer, it reverses inflammatory changes and abnormal proliferation caused by the imbalance of intestinal flora, resulting in lesions and the antitumor effect of the intestinal tract, improving the function of the intestinal barrier, regulating immunity, degrading carcinogenic compounds in the intestine, and inducing cancer cell death and antiproliferative effects [121,122]. The anti-cancer mechanisms of action of probiotics are anti-inflammatory and antioxidant, suggesting that they may improve disease management by promoting a healthy gut microbiome in the prevention and management of T2DM [123]. Furthermore, it reduces metabolic stress in T2D patients and has potential as an adjunct for the prevention and treatment of T2D by improving carbohydrate metabolism, fasting blood glucose, insulin sensitivity, and antioxidant status [124]. There is also recent research suggesting that probiotics may be involved in reducing the risk of developing Alzheimer’s disease and improving cognitive function. In AD, which is characterized by cognitive and memory impairment in the presence of Aβ and neurofibrillary tangles (NFTs), alterations in gut microbiota diversity and defects in the gut–brain axis have been linked to AD [125]. This can be approached by improving neurocognitive function and gastrointestinal symptoms, which are potential mechanisms of probiotics. Probiotics improve gastrointestinal symptoms of abdominal pain, bloating, and constipation in patients by strengthening the intestinal wall and inhibiting the adhesion of pathogenic bacteria [126]. They also use human milk oligosaccharides (HMOs) as a substrate to break down metabolites such as acetic acid, and SCFAs and bacterial metabolites act on antigen-presenting cells (APCs) and intestinal epithelia cells (IECs) through G protein-coupled receptors (GPCRs). This leads to a reduction in systemic inflammation of pro- and anti-inflammatory cytokines through the nuclear factor kappa-light chain enhancer of activated B cells, (NF-κB), and mitogen-activated protein kinase (MAPK) pathways [127,128]. The potential mechanisms by which probiotics act are illustrated in Figure 2.

Probiotics and vitamins also play unique roles in health maintenance. Probiotics support gut health, and vitamins contribute to cognitive well-being and a variety of physiological functions, which may be synergistic with probiotics [129]. The use of probiotics and vitamins has been shown to be safe [130], and no signs of toxicity have been observed, making them valuable as health substances for both children and elderly patients [131,132]. The interaction between nutrition and mental health is complex, especially in the gut [133]. Supplementing with probiotics and vitamins has been shown to increase anti-inflammatory response and total antioxidant capacity. This may result in decreased serum levels of high-sensitivity C-reactive protein, which has positive implications for AD treatment [134,135,136]. Probiotics have direct and indirect effects on brain function, primarily related to the hypothalamic–pituitary–adrenal (HPA) axis. Probiotics affect the HPA axis by modulating the ranges of corticosteroids and adrenocorticotropic hormone [137,138]. Probiotics also directly modulate central nervous system biochemistry by modifying the brain biochemicals 5-hydroxytryptamine (5-HT), neurotrophic factors, GABA, dopamine (DA), and c-Fos. Furthermore, probiotics are involved in gut–brain communication, which is affected by specific strains. The gut microbiome regulates metabolites such as short-chain fatty acids/SCFAs, exopolysaccharides/EPS, and tryptophan, which indirectly affect subsequent brain function. Your gut microbiome also works with your immune, endocrine, and nervous systems to influence your overall health. These actions suggest that probiotics may help improve brain function and balance the nervous system [139]. Huntington’s disease (HD), a neurodegenerative disease related to the structure and function of the brain, also transmits many microbial chemicals from the gut to the brain through the nervous system via the GBA. These affect brain physiology and are useful in the synthesis of neurotransmitters, immunology, and the metabolism of lipids and glucose. In addition, the anti-inflammatory and antioxidant effects of probiotics may slow the progression of Huntington’s disease [140].

The health benefits of probiotics are diverse and have been studied in the areas of anti-cancer, immune system, and cognitive function. This systematic review is important because it builds on recent research trends, but it has several limitations. First, we only included articles published in English. Second, our systematic review focused on probiotics that can be found in fermented foods, so we only analyzed probiotics that are microorganisms in food, excluding those that are a combination of probiotics and prebiotics, such as synbiotics. Therefore, future research should focus on probiotics and consider the specific types and combinations of probiotics. Furthermore, identifying the optimal dose and time of day for the health benefits of probiotics to be realized is an important challenge for clinical application today.

## 6. Conclusions

Fermented foods are an excellent model for establishing beneficial microbiota in the human body, recognizing the interaction with the microbiome to improve human health and nutrition. The first and most holistic approach to nutritional healthcare is nutrition from the human diet. Probiotic substances in fermented foods are beneficial bacteria and an important component of the human diet that can have a positive impact on health. The potential mechanisms of probiotics include antioxidant and anti-inflammatory effects, immune system modulation, and anti-cancer effects. Probiotics are recognized as a safe and beneficial strain of bacteria for human health and have a variety of health benefits, particularly for the digestive system, gastrointestinal tract, immune system, bones, obesity management, and nutrient absorption. In addition, it is used as a mainstay of chemotherapy and anti-cancer treatment for colorectal cancer, cognitive improvement in AD and HD, and important diseases such as inflammatory bowel disease and irritable bowel syndrome. Probiotics are beneficial strains for both physical and cognitive health based on the gut–brain axis, reducing TNF-α and IL through the NF-κB and MAPK pathways. They are also safe for use in children and the elderly.

Therefore, clinical trials should be conducted to determine the synergistic effects of various probiotics, postbiotics, probiotics, psychobiotics, and neobiotics in combination with prebiotics, as well as the specific dosage and duration. This could advance the possibility of using targeted therapeutics to address human diseases in the future.

## Data Availability

No new data were created or analyzed in this study. Data sharing is not applicable to this article.

## Abbreviations

VP—vitamin P; PI3K—phospholnositide-3 kinase; Akt—protein kinase B; ERK—extracellular signal-regulated kinase; PTEN—phosphatase and tensin homolog; PI3K-ATP—adenosine triphosphate; mTORC2—mechanistic target of rapamycin complex 2; GSK-3—synthase kinase-3; VEGF—vascular endothelial growth factor; STAT 3—signal transducers and activators of transcription 3; ROS—reactive oxygen species; PAL—phenylalanine ammonia lyase; C4H—cinnamic acid 4-hydroxylase; NF-kB—nuclear factor-kappa B; Nrf-2—nuclear factor erythroid 2-related factor 2; IL-6—interleukin 6; iNOS—inducible nitric oxide synthase; Q3G—quercetin-3-glucoside; LDL—low-density lipoprotein; PR—polylutein; NLRP3—nucleotide-binding domain-like receptor 3; NMIBC—non-muscle-invasive bladder cancer; PP—prickly pear; HRV—heart rate variability.
